# Depressive symptoms in individuals with family members requiring ADL assistance

**DOI:** 10.1186/s12199-019-0804-x

**Published:** 2019-07-15

**Authors:** Junhyun Kwon, Eun-Cheol Park, Woorim Kim, Dong-Woo Choi, Sung-In Jang

**Affiliations:** 1 Department of Public Health, Graduate School, Yonsei University, Seoul, Republic of Korea; 2 Institute of Health Services Research, Yonsei University, Seoul, Republic of Korea; 3 Department of Preventive Medicine, Yonsei University College of Medicine, 50 Yonsei-ro, Seodaemun-gu, Seoul, 120-752 Republic of Korea; 4 Department of Preventive Medicine, College of Medicine, Korea University, Seoul, Republic of Korea

**Keywords:** Depressive symptoms, Activities of daily living, Caregiving status, Family members

## Abstract

**Background:**

The number of patients with depressive symptoms worldwide is increasing steadily, and the prevalence of depression among caregivers is high. Therefore, the present study aimed to identify the effects of individuals’ caregiving status with respect to their family members requiring activities of daily living (ADLs) assistance on depressive symptoms among those aged 45 or over.

**Methods:**

Data were collected from the 2006–2016 using the Korean Longitudinal Study of Aging surveys. Participants were categorized into three groups based on their caregiving status with respect to family members requiring ADL assistance: whether they provided the assistance themselves, whether the assistance was provided by other caregivers, or whether no assistance was required. We analyzed the generalized estimating equation model and subgroups.

**Results:**

A total of 3744 men and 4386 women were included for the analysis. Men who cared for family members requiring ADL assistance had higher depressive symptoms than those with family members who did not require ADL assistance. Among women, participants who had family members requiring ADL assistance that they themselves or others were providing had higher depressive symptoms than those without family members requiring ADL assistance. Subgroup analysis was conducted based on age, job status, regular physical activities, participation status in social activities, and the number of cohabiting generations.

**Conclusions:**

The study results indicated higher depressive symptoms among those with family members requiring ADL assistance and those who care for such family members themselves. This suggests that an alternative to family caregiving is necessary, especially for the elderly, regardless of caregiver sex.

## Background

More than 300 million people worldwide have depression symptoms, and the number of depressed people increased by 18.4% between 2005 and 2015 [1, 2]. In South Korea, the number of depressed patients increased from 590,000 in 2012 to about 640,000 in 2016, an increase of over 50,000 people in just 4 years. An analysis of the number of depression patients by age group in 2016 showed that 55–59-year-olds had the highest rate of depression and that the overall depression distribution of middle-aged and senior individuals was generally high [3]. A complex interaction of psychological, social, or biological factors contributes to depression [4]. Various previous studies showed that the number of chronic health conditions [5, 6], cognitive impairment [7, 8], low exercise level [1, 9, 10], functional limitations [6, 9, 11], lack of social support [12], negative life events [13, 14], and other stressful events can affect depressive symptoms either directly or indirectly.

Among diverse stressful events, one of the factors that cause depression is caregiving provision to family patients [15, 16]. Particularly, the presence of family members requiring help in daily living can affect caregivers both physically and mentally. In fact, reports have shown that family caregivers often experience depressive symptoms when providing care for family members in terms of help in daily living due to various conditions [17, 18]. Previous studies on activities of daily living (ADL) patients and depression have indicated a high correlation between the two factors [19–21] and correspondingly, other studies have examined the mental health of caregivers who provide care for ADL patients [22, 23]. Among the related studies reported using the same KLoSA data as in this study, the degree of depression of a cohabiting spouse was also determined depending on the level of cognitive abilities of the spouse in need of care [24]. Additionally, family caregiving negatively affects not only the health but also the level of life satisfaction of caregivers [25], with the level of influence showing differences according to sex.

Therefore, this study examined the level of depressive symptoms of family caregivers according to caregiver sex by dividing the participants into three different groups according to their caregiving status. Specifically, caregiving status was categorized as the following: (1) individuals who help and provide care to family members who require ADL assistance by themselves, (2) individuals who have family members who are provided care and ADL assistance by other caregivers, and (3) individuals who have no family members requiring ADL assistance. The ADL criteria for this study were patients who required assistance with at least one of the categories of ADL assessment (feeding, dressing, grooming, physical ambulation, showering, and personal hygiene).

## Methods

### Study population

This study was carried out using data from the Korean Longitudinal Study of Aging (KLoSA) for the years 2006, 2008, 2010, 2012, 2014, and 2016. The KLoSA is a longitudinal study by the Korean Labor Institute targeting Korean individuals aged 45 years or older in households selected by multistage-stratified probability sampling (based on geographical area) to be representative of the nation. The surveys are carried out biennially through a computer-assisted personal interviewing technique and cover topics including demographics, family and social networks, physical and mental health, employment and retirement, income, and wealth.

There were a total 10,254 individual participants in the first baseline survey in 2006. Among these participants, 760 individuals had family members who need help regarding ADLs. The 2008 follow-up survey data contained 8688 individuals, the 2010 follow-up survey 7920 individuals, the 2012 follow-up survey 7486 individuals, the 2014 follow-up survey 7949 individuals (including 920 newly added participants), and the 2016 follow-up survey 7490 individuals (including 872 newly added participants).

Participants who had missing data for the variables used in this study were excluded. Hence, we excluded participants who were already receiving drugs for depression or for symptoms of depression. This led to the final inclusion of 8130 participants, including 3744 males and 4386 females, in the 2006 baseline study population.

### Measures

#### Depressive symptoms

The purpose of this study was to determine whether there is a difference in the Center for Epidemiological Studies Depression scale (CES-D 10), an indicator of the degree of depression, based on caregiver status with respect to a family member requiring ADL assistance. Depressive symptoms were assessed using CES-D 10 developed by the Boston site of Established Populations for Epidemiological Studies of the Elderly [26]. The CES-D 10 has 10 items that can be answered in a “Yes” or “No” format, and the total score is between zero and 10. On the CES-D 10, a total score of 4 or above is considered indicative of depression. Its validity as a test tool for older adults has been evaluated and proven [27, 28], and the Korean version of the CES-D has also been validated in the Korean population (Cronbach’s *α* = 0.909) [29].

#### Caregiving status and activities of daily living (ADLs)

ADLs can be divided into two areas: basic ADLs and instrumental ADLs (IADLs). ADLs require basic skills and focus on activities to take care of one’s own body. They include personal hygiene, continence management, dressing, feeding, and ambulating [30, 31]. IADLs include companionship and mental support, transportation and shopping, preparing meals, managing a person’s household, managing medications, communicating with others, and managing finances [32, 33]. In this study, we focused on basic ADLs and classified categories according to whether family members face difficulties in daily living. We divided the individuals into three categories according to their caregiving status with respect to their family members requiring ADL assistance: “Yes, by others,” “Yes, by myself,” and “No family members requiring ADL assistance.”

#### Covariates

Sociodemographic and health-related covariates were included in the present study. The included covariates were age (45–54, 55–64, 65–74, and ≥ 75), education level (elementary school or less, middle school, high school, university, or beyond), region (metropolitan, rural), working status (working or non-working), equalized household income (low, mid-low, mid-high, high), perceived health status (healthy, average, unhealthy), regular physical activities status (yes or no), participation status in social activities (yes or no), alcohol intake status (yes or no), smoking status (ever or never), the number of chronic diseases (none, 1, ≥ 2), and the number of cohabiting generations (couple, two generations, more than two generations). In the case of participation status in social activities, “yes” indicated participating in at least one of the categories of religion, leisure, friendship, hometown alumni meetings, and volunteering, and “no” indicated not participating in any of them.

### Statistical analysis

The general characteristics of the study participants were examined using *t* tests and analysis of variance (ANOVA) to compare differences in CES-D 10 scores and standard deviations. In analyzing the association between caregiving status of family members who need help with ADLs and depression scores, the generalized estimating equation (GEE) regression model, which is an extension of the quasi-likelihood approach used to analyze longitudinal correlated data, was used. The KLoSA was used in this study because it is longitudinal and contains yearly repeated measurements of the same individuals based on the first baseline data. Individuals originally included in the 2006 baseline year and their CES-D 10 scores were continuously followed up and measured in 2008, 2010, 2012, 2014, and 2016. Subgroup analysis was conducted based on age, job status, regular physical activities, participation status in social activities, and the number of cohabiting generations to investigate the influence of these factors on the relationship between family members’ ADL status and depressive symptoms. Analysis was stratified by sex to visualize whether different patterns exist between men and women. Since previous studies have shown that women experience a higher burden than men in similar caregiving situations [34–36], it is expected that there will be sex differences regarding the care of family members in this study as well. The calculated *P* values in the present study were all two-sided and considered significant at *P* < 0.05. All the analyses were carried out using the SAS software version 9.4 (SAS Institute, Cary, NC, USA).

## Results

Participants’ characteristics as recorded for the first wave of the study are presented in Table 1. The 8130 total participants were categorized into 3744 men and 4386 women. The total mean CES-D 10 scores of men and women were 2.285 ± 2.178 and 2.758 ± 2.441, respectively. Among the 3744 male participants, 170 (4.5%) individuals had family members who were provided care and ADL assistance by other caregivers, 84 (2.2%) individuals helped and provided care to family members who require ADL assistance by themselves, and 3490 (93.2%) individuals did not have family members requiring ADL assistance. The mean CES-D 10 scores were 2.171 ± 1.979 for the group with family members who were provided care and ADL assistance by other caregivers, 2.774 ± 2.456 for the group who helped and provided care to family members who require ADL assistance by themselves, and 2.279 ± 2.179 for the group who did not have family members requiring ADL assistance.

**Table 1:** Baseline characteristics of the study population by CES-D 10

Variables	Men					Women				
	Subjects		CES-D 10			Subjects		CES-D 10		
	*N*	%	Mean ± S.D		*P* value	*N*	%	Mean ± S.D		*P* value
Caregiving Status of family members requiring ADL assistance					0.2293					0.0129
Yes, by others*	170	4.5	2.17	1.98		222	5.1	2.81	2.45	
Yes, by myself	84	2.2	2.77	2.46		124	2.8	3.57	2.63	
No family members requiring ADL assistance	3490	93.2	2.28	2.18		4040	92.1	2.73	2.43	
Age					0.0064					< 0.0001
45–54	1280	34.2	1.91	1.87		1530	34.9	2.07	1.97	
55–64	1084	29.0	2.04	2.01		1226	28.0	2.55	2.39	
65–74	994	26.6	2.65	2.38		1023	23.3	3.36	2.54	
≥ 75	386	10.3	3.28	2.58		607	13.8	3.91	2.78	
Education level					0.0105					0.0643
Elementary school or less	1134	30.3	2.91	2.48		2414	55.0	3.29	2.65	
Middle school	644	17.2	2.30	2.24		740	16.9	2.40	2.11	
High school	1316	35.2	2.02	1.94		1011	23.1	2.00	1.93	
University or beyond	650	17.4	1.73	1.69		221	5.0	1.69	1.65	
Region					0.6862					0.7522
Metropolitan	1666	44.5	2.21	2.10		2010	45.8	2.66	2.41	
Rural	2078	55.5	2.34	2.24		2376	54.2	2.84	2.47	
Working Status					< 0.0001					0.1976
Working	2302	61.5	1.93	1.88		1154	26.3	2.35	2.22	
Non-working	1442	38.5	2.85	2.48		3232	73.7	2.90	2.50	
Equalized household income					0.0653					< 0.0001
Low	806	21.5	2.86	2.46		1228	28.0	3.45	2.66	
Mid-low	907	24.2	2.51	2.33		1124	25.6	2.95	2.52	
Mid-high	1207	32.2	2.17	2.08		1245	28.4	2.34	2.16	
High	824	22.0	1.65	1.62		789	18.0	2.06	2.05	
Perceive health status					< 0.0001					< 0.0001
Healthy	2187	58.4	1.80	1.72		2003	45.7	1.95	1.84	
Average	940	25.1	2.46	2.22		1291	29.4	2.77	2.35	
Unhealthy	617	16.5	3.75	2.79		1092	24.9	4.24	2.79	
Regular physical activities					< 0.0001					< 0.0001
Yes	1600	42.7	1.95	1.94		1566	35.7	2.26	2.13	
No	2144	57.3	2.54	2.31		2820	64.3	3.03	2.56	
Participation in social activities					< 0.0001					0.0061
Yes	882	23.6	2.91	2.63		1233	28.1	3.27	2.73	
No	2862	76.4	2.10	1.98		3153	71.9	2.56	2.29	
Smoking					0.2481					0.0028
Ever	2296	61.3	2.34	2.22		136	3.1	3.94	3.09	
Never	1448	38.7	2.20	2.11		4250	96.9	2.72	2.41	
Alcohol intake					0.2677					0.4269
Yes	2425	64.8	2.14	2.06		806	18.4	2.58	2.37	
No	1319	35.2	2.54	2.35		3580	81.6	2.80	2.46	
Number of chronic diseases					0.7316					0.0447
None	2183	58.3	2.03	1.97		2333	53.2	2.31	2.18	
1	1039	27.8	2.50	2.30		1269	28.9	2.96	2.52	
≥ 2	522	13.9	2.93	2.53		784	17.9	3.75	2.69	
Number of cohabiting generations					0.6453					0.4660
Couple	1598	42.7	2.50	2.30		1919	43.8	2.96	2.50	
Two generations	1784	47.7	2.06	2.01		1926	43.9	2.47	2.29	
Over two generations	362	9.7	2.44	2.31		541	12.3	3.07	2.62	
Total	3744	100	2.29	2.18		4386	100	2.76	2.44	

Other caregivers include other family members acting as informal caregivers as well as formal/professional individuals

With regard to the 4386 women in this study, 222 (5.1%) had family members who were provided care and ADL assistance by other caregivers, 124 (2.8%) provided the care themselves, and 4040 (92.1%) did not have family members requiring ADL assistance. The mean CES-D 10 scores were 2.806 ± 2.452 for the group with family members who were provided care and ADL assistance by other caregivers, 3.573 ± 2.632 for the group who helped and provided care to family members who require ADL assistance by themselves, and 2.730 ± 2.430 for the group who had no family members requiring ADL assistance.

Table 2 describes the relationship between CES-D 10 and the caregiving status of individuals with respect to family members requiring ADL assistance. Among men, those who had family members requiring ADL assistance for whom they provided care themselves had higher CES-D 10 scores (*β* 0.407, *P* = 0.0051) than those who did not have family members requiring ADL assistance. In women, those with family members requiring ADL assistance provided by other caregivers and those providing care themselves had higher depression scores (*β* 0.210, *P* = 0.0123 and *β* 0.506, P < 0.0001, respectively) than those who did not have family members requiring ADL assistance. In addition, in the case of women, the relationship between caregiving status of family members requiring ADL assistance and depressive symptoms showed a trend of increasing depressive symptoms in older age, and higher depression scores were obtained in the 65–74 years old group and the 75 years-or-older group (*β* 0.226, *P* = 0.0010 and *β* 0.082, *P* < 0.0001, respectively). In the case of men, regarding educational levels, those with lower educational levels tended to have more depressive symptoms than those in the university-or-beyond group (elementary school or less (*β*), 0.460; middle school (*β*), 0.272; high school (*β*), 0.164).

**Table 2:** Results of analysis of factors associated with CES-D 10 scores

Variables	Men		Women			
	*β*	S.E	*P* value	*β*	S.E	*P* value
Caregiving Status of family members requiring ADL assistance						
Yes, by others*	0.035	0.089	0.6947	0.210	0.084	0.0123
Yes, by myself	0.407	0.145	0.0051	0.506	0.125	< 0.0001
No family members requiring ADL assistance	Ref.			Ref.		
Age						
45–54	Ref.			Ref.		
55–64	− 0.003	0.053	0.9488	0.089	0.052	0.0836
65–74	0.051	0.071	0.4769	0.226	0.069	0.0010
≥ 75	0.122	0.090	0.1742	0.326	0.082	< 0.0001
Education level						
Elementary school or less	0.460	0.086	< 0.0001	0.364	0.114	0.0014
Middle school	0.272	0.090	0.0025	0.149	0.117	0.2032
High school	0.164	0.070	0.0194	− 0.041	0.106	0.7000
University or beyond	Ref.			Ref.		
Region						
Metropolitan	Ref.			Ref.		
Rural	0.168	0.053	0.0017	0.170	0.050	0.0007
Working Status						
Working	Ref.			Ref.		
Non-working	0.469	0.054	< 0.0001	0.255	0.047	< 0.0001
Equalized household income						
Low	Ref.			Ref.		
Mid-low	− 0.139	0.061	0.0213	− 0.321	0.054	< 0.0001
Mid-high	− 0.164	0.066	0.0134	− 0.467	0.058	< 0.0001
High	− 0.175	0.075	0.0194	− 0.411	0.066	< 0.0001
Perceive health status						
Healthy	− 1.540	0.069	< 0.0001	− 1.486	0.058	< 0.0001
Average	− 1.247	0.066	< 0.0001	− 1.158	0.053	< 0.0001
Unhealthy	Ref.			Ref.		
Regular physical activities						
Yes	− 0.282	0.041	< 0.0001	− 0.260	0.039	< 0.0001
No	Ref.			Ref.		
Participation in social activities						
Yes	− 0.407	0.056	< 0.0001	− 0.155	0.046	0.0008
No	Ref.			Ref.		
Smoking						
Ever	0.021	0.056	0.7097	0.663	0.142	< 0.0001
Never	Ref.			Ref.		
Alcohol intake						
Yes	− 0.222	0.051	<0.0001	− 0.118	0.059	0.0454
No	Ref.			Ref.		
Number of chronic diseases						
None	Ref.			Ref.		
1	0.007	0.055	0.8963	0.188	0.055	0.0006
≥ 2	0.176	0.072	0.0146	0.430	0.066	< 0.0001
Number of cohabiting generations						
Couple	− 0.009	0.082	0.9101	− 0.179	0.074	0.0154
Two generations	0.057	0.080	0.4767	− 0.018	0.073	0.8022
Over two generations	Ref.			Ref.		

*Other caregivers include other family members acting as informal caregivers as well as formal/professional individuals

The results of the analysis of the association between CES-D 10 and caregiving status of family members requiring ADL assistance stratified by age, working status, regular physical activities, participation in social activities, and the number of cohabiting generations are presented in Table 3. Among men, those who helped and provided care to family members who require ADL assistance by themselves showed statistically significant results in each subgroup. Regarding age, higher depression scores were observed in the 65–74 years old group (*β* 0.597, *P* = 0.0135) and the 75 years-or-older group (*β* 0.832, *P* = 0.0088). Regarding working status, higher depression scores were observed in the non-working group (*β* 0.686, *P* = 0.0011). Lastly, regarding regular physical activities, higher depression scores were observed among those not engaging in regular physical activities (*β* 0.444, *P* = 0.0222).

**Table 3:** Results of subgroup analysis of caregiving for ADL family members with CES-D 10

Variables			CES-D 10						
			No family members requiring ADL assistance	Yes, by others*			Yes, by myself		
			*β*	*β*	S.E	*P* value	*β*	S.E	*P* value
Men	Age	45–54	Ref.	− 0.151	0.191	0.4292	− 0.026	0.316	0.9356
		55–64	Ref.	0.191	0.147	0.1939	0.393	0.308	0.2030
		65–74	Ref.	− 0.034	0.169	0.8422	0.597	0.242	0.0135
		≥ 75	Ref.	0.316	0.237	0.1816	0.832	0.318	0.0088
	Working status	Working	Ref.	0.113	0.102	0.2681	0.075	0.189	0.6906
		Non-working	Ref.	− 0.010	0.161	0.9483	0.686	0.211	0.0011
	Regular physical activities	Yes	Ref.	− 0.004	0.123	0.9770	0.383	0.220	0.0812
		No	Ref.	0.042	0.121	0.7293	0.444	0.194	0.0222
	Participation in social activities	Yes	Ref.	− 0.006	0.095	0.9462	0.414	0.162	0.0105
		No	Ref.	5.236	1.970	0.0079	7.882	2.015	< 0.0001
	Number of cohabiting generations	Couple	Ref.	0.169	0.138	0.2194	0.651	0.248	0.0086
		Two generations	Ref.	− 0.266	0.124	0.0319	− 0.011	0.218	0.9612
		Over two generations	Ref.	− 3.399	1.117	0.0023	3.437	1.985	0.0834
Women	Age	45–54	Ref.	0.237	0.189	0.2095	0.421	0.262	0.1087
		55–64	Ref.	− 0.078	0.146	0.5936	0.518	0.227	0.0226
		65–74	Ref.	0.169	0.164	0.3013	0.923	0.277	0.0009
		≥ 75	Ref.	0.601	0.194	0.0019	0.113	0.247	0.6481
	Working status	Working	Ref.	0.143	0.126	0.2574	0.709	0.273	0.0093
		Non− working	Ref.	0.317	0.109	0.0037	0.473	0.142	0.0009
	Regular physical activities	Yes	Ref.	0.144	0.130	0.2668	0.809	0.224	0.0003
		No	Ref.	0.294	0.108	0.0067	0.454	0.151	0.0026
	Participation in social activities	Yes	Ref.	0.116	0.091	0.2044	0.651	0.147	< 0.0001
		No	Ref.	0.303	0.186	0.1031	0.053	0.243	0.8292
	Number of cohabiting generations	Couple	Ref.	0.157	0.113	0.1652	0.659	0.211	0.0018
		Two generations	Ref.	0.345	0.147	0.0189	0.275	0.185	0.1371
		Over two generations	Ref.	0.482	0.245	0.0492	0.783	0.284	0.0059

*Other caregivers include other family members acting as informal caregivers as well as formal/professional individuals

Among women, those who helped and provided care to family members who require ADL assistance by themselves showed statistically significant results in each subgroup. Regarding age, higher depression scores were observed in the 55–64 years old group (*β* 0.518, *P* = 0.0226) and the 65–74 years old group (*β* 0.923, *P* = 0.0009). Regarding working status, higher depression scores were observed in both the working (*β* 0.709, *P* = 0.0093) and the non-working (*β* 0.473, *P* = 0.0009) groups. Regarding the number of cohabiting generations, higher depression scores were observed in the more-than-two-generations group (*β* 0.783, *P* = 0.0059). Females with family members who were provided with care and ADL assistance by other caregivers also showed higher depression scores in the cohabiting-with-more-than-two-generations group (*β* 0.482, *P* = 0.0492).

## Discussion

After being adjusted for potential confounders, the results revealed that those individuals who helped and provided care to family members who require ADL assistance by themselves also showed higher depressive symptoms. In the case of men, those who provided ADL assistance to family members by themselves had higher depressive symptoms than those who did not have family members requiring ADL assistance. In the case of women, those with family members who were provided care and ADL assistance by other caregivers showed higher depressive symptoms as well.

In the subgroup analysis, age, working status, regular physical activities, participation in social activities, and the number of cohabiting generations were significantly associated with depressive symptoms in both men and women. In the case of men, depressive symptoms worsened for the age groups 65–74 years and ≥ 75 years if the participants were caring for family members requiring ADL assistance. In the case of women, depressive symptoms worsened for the age groups 55–64 years and 65–74 years if the participants were caring for family members requiring ADL assistance. In addition, among men, those who were cohabiting with spouses and engaging in caregiving by themselves were more likely to have depressive symptoms. Among women, those who were cohabiting with spouses or with more than two generations while caring for their family members requiring ADL assistance were more likely to have depressive symptoms. Furthermore, our findings that participants who are living with and caring for spouses requiring ADL assistance have higher depressive symptoms, particularly in women, supports the results of previous studies showing that participants living with spouses with a greater degree of dementia exhibited higher depression scores [24].

The other findings from this study are also consistent with previous studies that ADL assistance is primarily required as a consequence of stroke or dementia that occur as a result of aging [37, 38]. Korea has become an aging society, with a population of over the age of 65 exceeding 14% in 2017, and it is expected that the size and proportion of the population aged 80 and above will increase at a faster pace [39]. It can be predicted that, over time, the proportion of the population with ADL dependence will increase due to the chronic diseases caused by the increase in the proportion of elderly people. As reported in this study, depression was found to be high in both men and women when providing ADL assistance to family members. In women, those with family members requiring ADL assistance provided by other caregivers showed higher depressive symptoms, inferring that women are more likely to feel burdensome and, as a result, become more vulnerable to depression than men [34, 36, 40].

Previous studies have shown that high depressive symptoms and negative life satisfaction were associated with caring for a spouse or elderly family members [25, 41–43]. Considering that caregivers are generally middle-aged or older, such depressive symptoms and negative life satisfaction affecting these caregivers may be a sufficient risk factor for future physical illnesses, such as a declining competence in daily living and falls [44, 45]. On the other hand, it has also been reported that receiving care at home rather than in a nursing home is more effective for managing depressive symptoms and life satisfaction in patients [46]. Since the majority of ADL patients are, therefore, cared for by family members, the risk of depressive symptoms for caregivers needs particular attention. In Korea, depressive symptoms observed in family caregiving situations are particularly important because about 72% of patients with dementia are cared for by family members [47]. Therefore, appropriate support and measures are needed to reduce depressive effects among individuals who are cohabiting while family members receive care at home.

The KLoSA uses well-established data that has been conducted on middle-aged or older individuals, but it has some limitations. First, through the use of these data, we were unable to identify the extent of support required for the family members requiring ADL assistance. However, the study offers general statistical results. Second, we could not determine if other family members besides the study participants or external caregivers were providing ADL assistance for those individuals categorized as having family members who were being provided ADL assistance. Third, ADL is mainly used for evaluating dementia [48, 49], and it is also an important criterion for judging the effectiveness of dementia treatment [50]. In other words, ADL is generally an indicator of evaluation related to dementia. However, in the KLoSA questionnaire, it was unclear what caused participants’ family members to require ADL assistance. Fourth, the number of participants who helped their family members with ADL assistance was relatively small. Fifth, there are other factors that can affect the depression levels of middle-aged and senior individuals in addition to those incorporated in this study. However, due to data limitations, we were unable to consider all such potential causes. Finally, we could not clarify how many family members needed assistance in each family. Depending on the number of cohabiting generations, in the case of “couple,” we may know how many family members needed assistance, but if individuals belonged to the “two generations” or “more than two generations” group, we were not able to confirm this information.

To further develop the study findings, a variety of systems and further studies are needed on the mental health of the caregiver.

## Conclusions

The results of this study indicate that both men and women have higher depressive symptoms among those with family members requiring ADL assistance and those who care for such members themselves than those who do not have family members requiring ADL assistance. In addition, there are also significant associations showing that among those who care for family members requiring ADL assistance, depressive symptoms tended to increase with their ages. Therefore, it is important that an alternative to family caregiving is necessary, especially for the elderly.

## Data Availability

This study’s data are generated and analyzed from the Korean Longitudinal Study of Aging (KLoSA) and these longitudinal data are available from the Korean Labor Institute ( https://survey.keis.or.kr/index.jsp).

## Abbreviations

ADL: Activities of daily living
CES-D: The Center for Epidemiological Studies Depression scale
GEE: Generalized estimating equation
IADL: Instrumental activities of daily living
KLoSA: The Korean Longitudinal Study of Aging
