# Correction to: Depressive symptoms in individuals with family members requiring ADL assistance

**DOI:** 10.1186/s12199-019-0809-5

**Published:** 2019-08-28

**Authors:** Junhyun Kwon, Eun-Cheol Park, Woorim Kim, Dong-Woo Choi, Sung-In Jang

**Affiliations:** 1 Department of Public Health, Graduate School, Yonsei University, Seoul, Republic of Korea; 2 Institute of Health Services Research, Yonsei University, Seoul, Republic of Korea; 3 Department of Preventive Medicine, Yonsei University College of Medicine, 50 Yonsei-ro, Seodaemun-gu, Seoul, 120-752 Republic of Korea; 4 Department of Preventive Medicine, College of Medicine, Korea University, Seoul, Republic of Korea

**Correction to: Environ Health Prev Med (2019) 24:49**

**https://doi.org/10.1186/s12199-019-0804-x**

Following publication of the original article [1], the authors reported an error in Table 2 in their paper. The table entry “Men’s P value” was mistakenly included under the table heading “Women”. The original article [1] has been updated.

The correct Table 2 is shown below.

**Table 2:** Results of analysis of factors associated with CES-D 10 scores

Variables	Men			Women		
	β	S.E	*P*-value	β	S.E	*P*-value
Caregiving Status of family members requiring ADL assistance						
Yes, by others^a^	0.035	0.089	0.6947	0.210	0.084	0.0123
Yes, by myself	0.407	0.145	0.0051	0.506	0.125	<.0001
No family members requiring ADL assistance	Ref.			Ref.		
Age						
45–54	Ref.			Ref.		
55–64	−0.003	0.053	0.9488	0.089	0.052	0.0836
65–74	0.051	0.071	0.4769	0.226	0.069	0.0010
≥ 75	0.122	0.090	0.1742	0.326	0.082	<.0001
Education level						
Elementary school or less	0.460	0.086	<.0001	0.364	0.114	0.0014
Middle school	0.272	0.090	0.0025	0.149	0.117	0.2032
High school	0.164	0.070	0.0194	−0.041	0.106	0.7000
University or beyond	Ref.			Ref.		
Region						
Metropolitan	Ref.			Ref.		
Rural	0.168	0.053	0.0017	0.170	0.050	0.0007
Working Status						
Working	Ref.			Ref.		
Non-working	0.469	0.054	<.0001	0.255	0.047	<.0001
Equalized household income						
Low	Ref.			Ref.		
Mid-low	−0.139	0.061	0.0213	−0.321	0.054	<.0001
Mid-high	−0.164	0.066	0.0134	−0.467	0.058	<.0001
High	−0.175	0.075	0.0194	−0.411	0.066	<.0001
Perceive health status						
Healthy	−1.540	0.069	<.0001	−1.486	0.058	<.0001
Average	−1.247	0.066	<.0001	−1.158	0.053	<.0001
Unhealthy	Ref.			Ref.		
Regular physical activities						
Yes	−0.282	0.041	<.0001	− 0.260	0.039	<.0001
No	Ref.			Ref.		
Participation in social activities						
Yes	−0.407	0.056	<.0001	−0.155	0.046	0.0008
No	Ref.			Ref.		
Smoking						
Ever	0.021	0.056	0.7097	0.663	0.142	<.0001
Never	Ref.			Ref.		
Alcohol intake						
Yes	−0.222	0.051	<.0001	−0.118	0.059	0.0454
No	Ref.			Ref.		
Number of chronic diseases						
None	Ref.			Ref.		
1	0.007	0.055	0.8963	0.188	0.055	0.0006
≥ 2	0.176	0.072	0.0146	0.430	0.066	<.0001
Number of cohabiting generations						
Couple	−0.009	0.082	0.9101	−0.179	0.074	0.0154
Two generations	0.057	0.080	0.4767	−0.018	0.073	0.8022
Over two generations	Ref.			Ref.		

^a^Other caregivers include other family members acting as informal caregivers as well as formal/professional individuals
